# The Arctic Soil Bacterial Communities in the Vicinity of a Little Auk Colony

**DOI:** 10.3389/fmicb.2016.01298

**Published:** 2016-09-09

**Authors:** Sylwia Zielińska, Dorota Kidawa, Lech Stempniewicz, Marcin Łoś, Joanna M. Łoś

**Affiliations:** ^1^Department of Molecular Biology, University of GdanskGdansk, Poland; ^2^Department of Vertebrate Ecology and Zoology, University of GdanskGdansk, Poland

**Keywords:** bacterial community structure, Arctic soil, 16S rRNA gene, NGS, *Alle alle*

## Abstract

Due to deposition of birds' guano, eggshells or feathers, the vicinity of a large seabirds' breeding colony is expected to have a substantial impact on the soil's physicochemical features as well as on diversity of vegetation and the soil invertebrates. Consequently, due to changing physicochemical features the structure of bacterial communities might fluctuate in different soil environments. The aim of this study was to investigate the bacterial assemblages in the Arctic soil within the area of a birds' colony and in a control sample from a topographically similar location but situated away from the colony's impact area. A high number of OTUs found in both areas indicates a highly complex microbial populations structure. The most abundant phyla in both of the tested samples were: *Proteobacteria, Acidobacteria, Actinobacteria*, and *Chloroflexi*, with different proportions in the total share. Despite differences in the physicochemical soil characteristics, the soil microbial community structures at the phylum level were similar to some extent in the two samples. The only share that was significantly higher in the control area when compared to the sample obtained within the birds' colony, belonged to the *Actinobacteria* phylum. Moreover, when analyzing the class level for each phylum, several differences between the samples were observed. Furthermore, lower proportions of *Proteobacteria* and *Acidobacteria* were observed in the soil sample under the influence of the bird's colony, which most probably could be linked to higher nitrogen concentrations in that sample.

## Introduction

The high Arctic archipelago of Svalbard, with Spitsbergen being the largest island, is a breeding ground for many seabirds, including the little auk (*Alle alle*) which is considered to be the most numerous seabird species in the Palearctic. The little auk population in Hornsund exceeds one million members and is one of the largest colonies in the world (Isaksen, 1995). Birds arrive at this colony area in large numbers in mid-April and stay there until late August (Stempniewicz, 2001). Marine birds that forage at sea and breed on land deposit large amounts of guano, eggshells, feathers, and carcasses near their colonies. This large-scale transport of organic and inorganic matter from the sea to the land is crucial for many Arctic and Antarctic terrestrial ecosystems (Stempniewicz et al., 2007; Zwolicki et al., 2015). It was previously shown that the guano deposition influences various physical and chemical soil parameters and enhances the formation of ornithogenic soils, thereby facilitating the development of associated terrestrial plant communities (Stempniewicz et al., 2007; Zwolicki et al., 2013; Wojciechowska et al., 2015). It has been also shown that abundance of the soil invertebrates, such as springtails (*Collembola*) or water bears (*Tardigrada*), is higher in the areas of ornithogenic enrichment of the soil (Zawierucha et al., 2015; Zmudczyñska-Skarbek et al., 2015b). There are several studies denoting a considerable impact of the large seabird colonies on the soil properties in the nutrient-deprived Arctic ecosystems (e.g., Croll et al., 2005; Zwolicki et al., 2013, 2015; Skrzypek et al., 2015). Similarly, birds' contribution to the fertilization of the coastal waters in the vicinity of the colonies has also been denoted (Zelickman and Golovkin, 1972; Zmudczyñska-Skarbek et al., 2015a). Nonetheless, the influence of bird-derived soil fertilization on the structure and abundance of microorganisms has not been reported so far in the Arctic environment. Since the little auks may have a substantial impact on the soil environment, diversity in the Arctic soil bacterial communities within this colony's vicinity could be expected.

Due to harsh environmental conditions, microbial communities in the Arctic soil were expected to be comprised of a relatively low number of bacterial species, but in fact they are as diverse as those found in other biomes (Neufeld and Mohn, 2005; Koyama et al., 2014). Diversity of the Arctic soil bacterial communities can be influenced by various natural factors. The vicinity of a large breeding colony of planktivorous seabirds, the little auks, seems to have a substantial impact on the soil environment (Zwolicki et al., 2013). It was also reported that the ornithogenic soils which arose in the areas of the Adèlie Penguin rookeries in the Ross Sea region of the Antarctica, are characterized with a higher microbial biomass than the mineral soils of the Ross Sea region where the penguins were absent. These soils are also characterized by a higher organic carbon content and higher total nitrogen and phosphorus, with high electrical conductivity and large variations in pH (Aislabie et al., 2009). Likewise, at the King George Island, the seabird colonies were reported to affect the soil properties and vegetation (Zwolicki et al., 2015). Therefore, the aim of this study was to compare, by using the Next Generation Sequencing (NGS) in 16S rRNA gene analysis, the bacterial communities in the soil samples originating from two areas: (1) in the vicinity of a seabird colony and (2) not influenced by seabirds. The samples being compared were collected from topographically similar locations. Since bacterial communities might vary due to external factors affecting the soil environment such as physicochemical conditions, different bacterial communities may be expected in each soil sample. These differences might span the entire community structure, or be limited only to the single phyla present.

## Materials and methods

### Study area and sample collection

**Figure 1:**
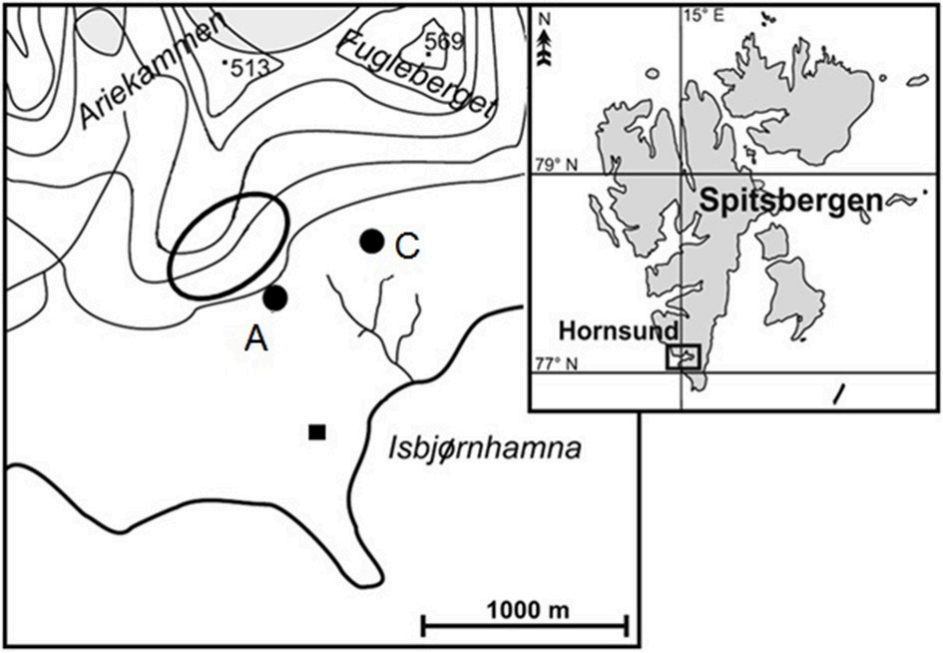
**Maps of the study area**. **Right:** Location of the Hornsund fiord at the Spitsbergen. **Left:** Close-up of the north part of the Hornsund fiord with Ariekammen and Fugleberget; black square—the Polish Polar Station, black oval frame—the little auk colony, black spots—soil sampling sites: A—the area within the little auks colony, and C—the control area away from the routine flight route of the seabirds.

Soil samples were collected in the north part of the Hornsund fiord (SW Spitsbergen; 77° 0′ N, 15° 33′ E) at the beginning of August 2013 (Figure 1). One of the samples (A)uk was collected in the area influenced by a large breeding colony of planktivorous little auks down the slope, at the edge of the colony. This colony was situated on the Ariekammen mountain slope and consisted of ca. 15,000 breeding pairs (Puczko and Stempniewicz, 2013). Lush vegetation, with predominating *Deschampsia alpina, Prasiola crispa, Cerastium arcticum*, and *Chrysosplenium tetrandum* covered ca. 95% of this well-fertilized area (Zmudczyñska et al., 2012). Sample (C)ontrol was collected in a topographically similar location (ca 550 m from the sample A), but away from the routine seabird flight route, hence experiencing only a negligible ornithogenic impact. Vegetation in the control area had a much lower total coverage (ca. 10%) and was almost entirely represented by a *Sanionia uncinata-Salix polaris* community (Zmudczyñska et al., 2012). Both samples were collected from the upper 15–20 cm of the soil, frozen 0.5 h after collection and stored at −20°C until further analysis.

### Physical and chemical analysis of the soil samples

Soil physicochemical features were measured according to methodology described by Zwolicki et al. (2013). The soil dry mass (%) was assessed, as well as the soil conductivity (μS cm^−1^), pH and nitrogen (${\text{NO}}_{3}^{-}$ and ${\text{NH}}_{4}^{+}$) and phosphate (${\text{PO}}_{4}^{3-}$) content.

### DNA extraction and bacterial 16S rRNA gene amplification

After thawing, the DNA was extracted from each soil sample (extraction done in triplicate for each sample) using the FastDNA® SPIN Kit for Soil and the FastPrep® Instrument (MP Biomedicals, Santa Ana, CA). To avoid cross contamination of the samples, the whole process was performed using sterile equipment. The quantity and quality of the extracted DNA was evaluated by using a Nano Drop spectrophotometer followed by agarose gel electrophoresis. The DNA was stored at −20°C until further use.

The V3-V4 hypervariable regions of bacterial 16S rRNA gene were amplified, using the following primer pair: 341F-CCTACGGGNGGCWGCAG and 785R-GAC TACHVGGGTATCTAATCC. The targeted gene regions have been shown to be the most suitable for Illumina sequencing (Klindworth et al., 2013). Each sample was amplified with NEBNext® High-Fidelity 2xPCR Master Mix (New England BioLabs) according to the manufacturer's instructions. Paired-end (PE, 2 × 250 nt) sequencing was performed with an Illumina MiSeq (MiSeq Reagent kit v2) by the Genomed company (Warsaw, Poland) and following manufacturer's run protocols (Illumina, Inc., San Diego, CA, USA).

### Sequencing data and statistical analyses

The data obtained for the 3 replicates of the DNA extraction for each soil sample were merged and considered as one sample in further taxonomic analysis. The intention of this procedure was to obtain a more reliable view of the bacterial communities' structure. The samples were processed and analyzed by using the Quantitative Insights Into Microbial Ecology (Qiime) pipeline v 1.8.0 software (Caporaso et al., 2010). Low quality paired end (PE) reads were discarded from further analysis and the quality-filtered reads were merged based on the overlap of the PE read with the use of fastq-joint (Aronesty, 2011). The remaining sequences that did not meet the quality criteria were removed from further analysis. Clustering of the operational taxonomic units (OTUs) at the 97% similarity was performed by using the uclust method (Edgar, 2010). OTUs were assigned to taxa by using GreenGenes v13_5 as a reference (McDonald et al., 2012). The chimera sequences were detected with the use of the Chimera Slayer tool (Haas et al., 2011). After sequence count normalization, statistical analysis was performed on the bacterial and archaeal composition. The diversity indices were estimated based on clusters, including the Chao1, Shannon, and Simpson indices. The NGS data are deposited and fully available under the following study accession numbers: PRJEB9513and PRJEB9514, at ENA—the European Nucleotide Archive.

The pairwise comparison of the two soil samples A and C, was done using the SIMPER (Similarity Percentage) method based on the Bray-Curtis similarity measure. SIMPER is a simple method for assessing which phyla are primarily responsible for an observed difference between samples (Clarke, 1993). The Bray-Curtis similarity measure (multiplied by 100) is commonly used with SIMPER. Also, the Bray-Curtis similarity is a popular similarity index used for abundance data (Bray and Curtis, 1957). The overall average dissimilarity was computed using all of the phyla, while the phylum-specific dissimilarities were computed for each phylum individually. The chi-square test was performed to examine the differences between the samples, and the samples were compared considering the phylum level. All analyses were done using the PAST 3.0 software (Hammer et al., 2001).

## Results

### Physical and chemical soil features

Clear differences in the measured ion concentration and soil conductivity, as well as in the soil dry mass, could be seen between samples A and C (Table 1), as it was previously reported by Zwolicki et al. (2013). Sample A showed much higher content of phosphates (over 12-fold) and nitrates (over 7-fold) when compared to sample C. The pH of sample A was lower when compared to sample C (6.51 and 6.74 respectively).

**Table 1:** **Values for the soil ion content (mg/kg soil dry mass), pH, conductivity (mS/cm) and dry mass (%) for samples A and C**.

**Parametres/Sample ID**	**A**	**C**
${\text{PO}}_{4}^{3-}$	23.61	1.842
${\text{NO}}_{3}^{-}$	43.18	5.768
${\text{NH}}_{4}^{+}$	3.575	1.496
pH	6.51	6.74
Conductivity	113.9	61.7
Dry mass	66.28	97.64

### General description of the sequencing results

From the soil sample A, collected within the little auk colony, we obtained 230,253 good quality 16S rRNA gene sequences (V3-V4 region), while 279,122 gene sequences were obtained from the control sample C. At the phylum level, in case of both samples we were able to classify more than 99.97% of all of the obtained sequences. A detailed taxonomic analysis on different ranks, for both soil samples, is presented in sunburst charts (Presentation 1) and in a table (Supplementary Table 1). 3600 OTUs were obtained for sample A, and 4844 OTUs for sample C; both of the tested samples had 2822 OTUs in common. The Shannon index value for sample A was 8.92, and 9.31 for sample C, while the Chao1 index value was 4391 and 5496, respectively. The Simpson index value was 0.99 for both of the tested samples. The diversity indices represent a randomly selected subset for a sample normalized to 229,213 sequences. The analysis of sample similarity was done on the relatively high taxonomic level due to fact, that we were unable to identify microorganisms on lower taxonomic levels and unclassified microorganisms contributed even up to 80% of total community share. Additionally, our main goal was to present global analysis and differences between two locations with differences in soil features. The attempt to compare samples on lower phylogenetic level would result in a fragmented picture, not necessary reflecting the global difference in microbial communities.

### Microbial community composition

The analysis of bacterial communities found in the soil sample collected within the little auk colony showed that 99.99% of the total reads were affiliated with *Bacteria* and 0.01% with *Archaea* (Presentation 1). In the control soil sample, the total reads representing *Bacteria* constituted 99.96% and *Archea* 0.04% (Presentation 1). Taxonomy-based analysis of both samples indicated that the soil bacterial communities consisted of 34 phyla of which 32 were represented in both samples (Presentation 1, Supplementary Table 1). Sample A was characterized by a lack of the GN04 and Thermi phyla, while in sample C no *Lentisphaerae* and WS4 were found. The combined share of those phyla was less than 0.022% of the total community for each tested sample.

**Figure 2:**
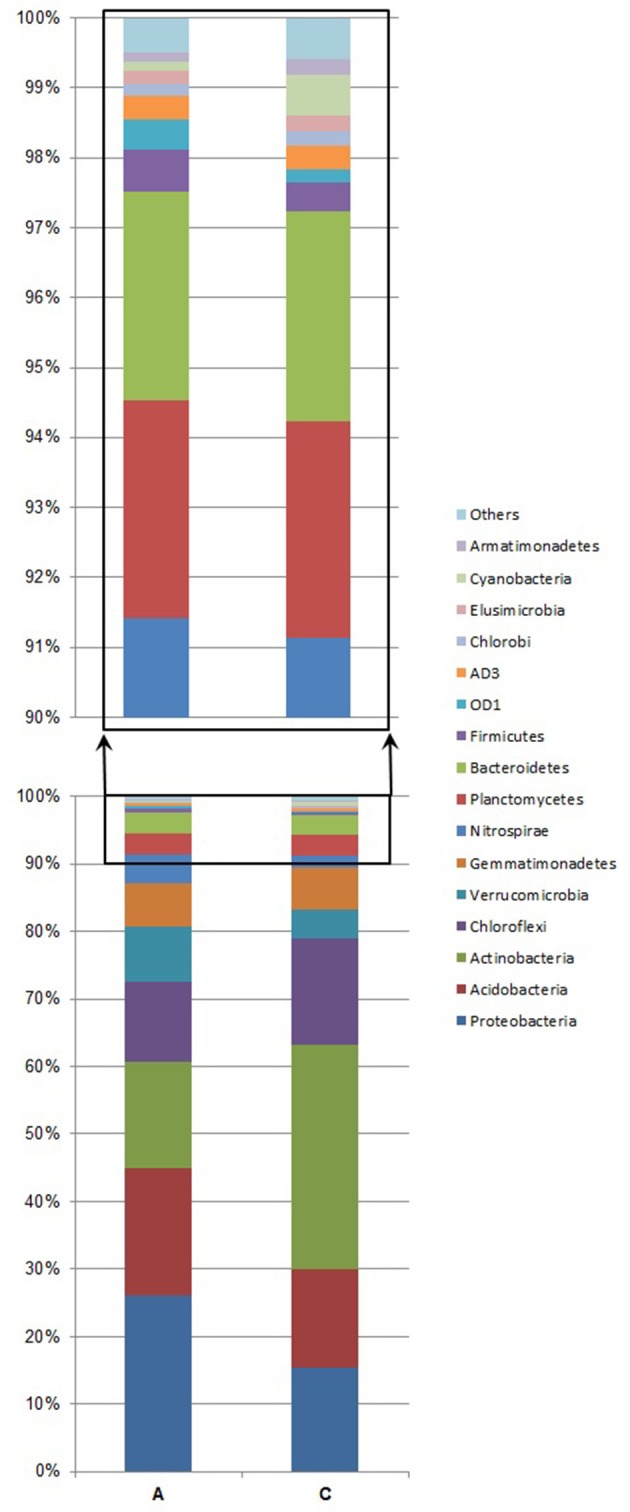
**Abundance of bacterial 16S rRNA gene sequences at the phylum level**. Analysis of microbial community structure in the soil sample under the little auk' colony influence (A) and in the area away from the routine flight route of birds, treated as a control sample (C). “Others” denotes the following: WS3, TM7, OP11, WS2, TM6, WPS-2, FBP, BRC1, WS4, BHI80-139, NKB19, *Fibrobacteres*, GN02, OP3, *Spirochaetes, Chlamydiae, Lentisphaerae*, PAUC34f, GN04, [Thermi], and unassigned.

The most abundant phyla found in sample A were: *Proteobacteria, Acidobacteria, Actinobacteria*, and *Chlorofelxi* (Figure 2, Table 2). Combined these phyla accounted for more than 72.5% of the total bacterial sequences obtained. Moreover, 16 of those phyla were responsible for 99.5% of the total bacterial community. In sample C, the most abundant phylum was *Actinobacteria*. Other phyla with a significant share were: *Chloroflexi, Proteobacteria*, and *Acidobacteria* (Figure 2, Table 2). These phyla jointly accounted for more than 79% of the total bacterial sequences obtained. In this case, 17 of those phyla were responsible for more than 99.5% of the total bacterial population. When comparing both samples, sample C contains almost twice as high abundance of *Actinobacteria* than sample A, whereas for the *Chloroflexi* phylum, the difference in abundance between the two samples was only minor. In the case of *Proteobacteria* and *Verrucomicrobia*, their higher abundance in the bacterial community structure is related to lower soil pH and higher nitrogen distribution. The share of *Actinobacteria* and *Proteobacteria* was the factor that mostly contributed to the differences between the samples (Table 2). Considering the share of each phylum in the total bacterial composition, the only significant differences between the two samples were observed when comparing the share of *Actinobacteria* (χ^2^ = 8.91, *df* = 1, *p* = 0.004). Furthermore, lower proportions of *Proteobacteria* and *Acidobacteria* were observed in the soil sample collected within the bird colony than in the control sample, however, these differences were found to be statistically insignificant (χ^2^ = 3.72, *df* = 1, *p* = 0.054 and χ^2^ = 0.60, *df* = 1, *p* = 0.44, respectively). Considering all phyla at once, the Bray-Curtis distance measure indicated 78% similarity between the two samples (A and C) and the chi-square test did not indicate a statistically significant difference between the samples (χ^2^ = 13.09, *df* = 36, *p* = 1.0).

**Table 2:** **Average dissimilarity between the sample A under the little auk colony and sample C as the control sample compared by SIMPER analysis based on the Bray-Curtis similarity measure at the phylum level**.

**Phylum**	**A (%)**	**C (%)**	**Av. dissimilarity**	**Contribution (%)**	**Cumulative (%)**
*Actinobacteria*	15.7	33.3	8.8	39.3	39.3
*Proteobacteria*	26	15.5	5.3	23.6	63
*Acidobacteria*	18.8	14.5	2.2	9.8	72.7
*Verrucomicrobia*	8.1	4.2	2	8.9	81.6
*Chloroflexi*	11.9	15.8	1.9	8.7	90.3
*Nitrospirae*	4.3	1.7	1.3	5.8	96
*Cyanobacteria*	0.1	0.6	0.2	1	97.1
*Gemmatimonadetes*	6.5	6.2	0.1	0.6	97.7
OD1	0.4	0.2	0.1	0.5	98.2
*Firmicutes*	0.6	0.4	0.1	0.4	98.7
*Armatimonadetes*	0.1	0.2	0	0.2	98.9
WS3	0.1	0.2	0	0.2	99.1
TM7	0.1	0.1	0	0.2	99.2
OP11	0.1	0	0	0.1	99.3
*Elusimicrobia*	0.2	0.2	0	0.1	99.5
WPS-2	0	0	0	0.1	99.5
*Bacteroidetes*	3	3	0	0.1	99.6
*Fibrobacteres*	0	0	0	0.1	99.6
Unknown	0	0	0	0	99.7
WS4	0	0	0	0	99.7
*Spirochaetes*	0	0	0	0	99.7
FBP	0	0	0	0	99.8
BRC1	0	0	0	0	99.8
*Chlorobi*	0.2	0.2	0	0	99.8
WS2	0.1	0	0	0	99.9
*Planctomycetes*	3.1	3.1	0	0	99.9
NKB19	0	0	0	0	99.9
BHI80-139	0	0	0	0	99.9
OP3	0	0	0	0	100
TM6	0.1	0	0	0	100
GN02	0	0	0	0	100
AD3	0.3	0.3	0	0	100
*Lentisphaerae*	0	0	0	0	100
*Chlamydiae*	0	0	0	0	100
GN04	0	0	0	0	100
PAUC34f	0	0	0	0	100
[Thermi]	0	0	0	0	100

**Figure 3:**
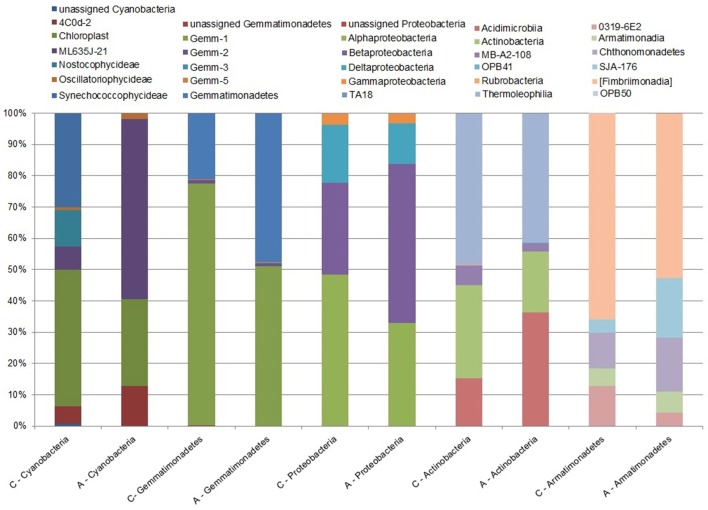
**Abundance of bacterial 16S rRNA gene sequences with significant differences between the tested samples at the class level**. The chi-square test, performed at the class level for soil sample A (within the little auk colony) and for sample C (treated as a control) indicates a significant difference between the samples among the following five phyla: *Actinobacteria, Armatimonadetes, Cyanobacteria, Gemmatimonadetes*, and *Proteobacteria*.

At the class level, the two samples differed significantly (χ^2^ = 191.64, *df* = 85, *p* < 0.0001). In sample A, the most abundant bacteria were: *Betaproteobacteria, Alphaproteobacteria, Chloracidobacteria*, and *Thermoleophilia*. In contrast, *Thermoleophilia, Actinobacteria, Alphaproteobacteria*, and *Chloracidobacteria* were the most dominant classes in sample C (Presentation 1, Supplementary Table 1). *Betaproteobacteria* constitute the highest portion in sample A, with their share amounting to more than 13%. However, we can observe in the control sample that their share is reduced and amounts to only 4.5% of the total population. Additionally, distribution of *Thermoleophilia* and *Actinobacteria* seems to be reduced in soils with lower pH and increased nitrogen ion concentration. At the class level, only *Alphaproteobacteria* share a similar proportion among the most abundant bacteria in both tested samples, despite the change in pH and availability of nitrogen. Additional chi-square test analysis, comparing samples A and C at the class level within each phylum separately, indicated significant differences between the samples for five phyla: *Actinobacteria, Armatimonadetes, Cyanobacteria, Gemmatimonadetes*, and *Proteobacteria* (Supplementary Tables 1, 2; Figure 3).

## Discussion

Seabird guano is one of the three major nitrogen sources in the polar terrestrial ecosystems, however the tundra fertilization by seabirds is unevenly distributed over time and space as a consequence of patchy distribution of bird colonies and their short breeding period. As was shown previously, deposition of nutrient-rich guano by little auks considerably changes the physicochemical parameters of the soil in the vicinity of the bird colony (Zwolicki et al., 2013). However, despite striking differences in physicochemical soil characteristics, as well as differences in vegetation structure and invertebrates abundance (Wojciechowska et al., 2015; Zawierucha et al., 2015; Zmudczyñska-Skarbek et al., 2015b), we did not find substantial differences at the phylum level of taxonomic ranks in the soil microbial communities in the two samples tested. The similarity between these two tested bacterial communities was 78%, and in addition, the chi-square test did not indicate a statistically significant difference between the samples tested. Still, *Actinobacteria* and *Proteobacteria* were responsible for the differences between the samples and further analysis considering each phylum separately indicated that significant differences between the tested samples were specifically found within the *Actinobacteria* phylum.

High number of OTUs, as well as high values of microbial diversity indexes (the Shannon's and Simpson's indexes), suggest a high number of species in both tested samples. Moreover, the high number of OTUs observed in both samples indicates that both soil microbial communities are highly complex. Rarefaction trends analysis indicated that the sampling of bacterial communities were close to complete, which indicates sufficient efficiency of the DNA extraction method (Supplementary Figure 1). Furthermore, we obtained 2822 OTUs that both samples have in common, which indicates a high similarity between the tested samples. However, there is also a large group of OTUs that are specific for each particular soil sample. Sample C is characterized by a higher number of OTUs. It seems that more alkaline conditions and impecunious nutrient content in the control area correspond to a more diversified microbiome composition.

As sample A origins from an area strongly influenced by seabirds, it could be expected to contain a less diverse and thus more specialized groups of bacteria than the control sample (Koyama et al., 2014). Still, the most abundant phyla, in both tested samples, were: *Proteobacteria, Acidobacteria, Actinobacteria*, and *Chloroflexi*, with varying proportion in the total share between the samples. Such abundance and composition of share in bacterial communities is common for most soil samples of different origin studied so far (Janssen, 2006), including the Arctic soil (Chu et al., 2010). Also, minor groups of the bacterial community structure were similar to other groups previously described for the Arctic soil (Chu et al., 2010; Kim et al., 2014). Until recently, microbial communities in the Arctic soil were expected to have a low level of bacterial species, but in fact they are characterized by an equivalent level of diversity as other biomes are (Koyama et al., 2014). Moreover, Chu et al. (2010) had demonstrated that the Arctic soil bacterial communities are characterized by similar levels of variability, richness and phylogenetic diversity, as soils obtained from a wide range of lower latitudes. This suggests a common diversity structure within soil bacterial communities around the globe. Furthermore, Arctic soil bacterial community structure is related to local environmental factors, especially with soil acidity, similarly as it was observed for the fungal community composition in this High Arctic region (Zhang et al., 2016).

It was previously demonstrated that the soil pH has a fundamental impact on the bacterial communities (Shen et al., 2013), thus, the lack of significant differences between the tested samples at higher taxonomic ranks may result from a minor dissimilarity between the soil pH values. Lower proportions of *Proteobacteria* and *Acidobacteria*, mostly responsible for the differences between the two samples (Table 2), were observed in the soil sample under the influence of the bird colony, characterized by higher acidity, whereas the share of *Actinobacteria* and *Chlorofelxi* was higher under more alkaline conditions, i.e., in the control area (Figure 2). According to our observations, the abundance of *Proteobacteria* was lower under more alkaline conditions, although abundance of *Alphaproteobacteria* was similar in both tested soil samples. We also observed a large disproportion in the contribution of *Betaproteobacteria* in the total share of bacterial communities in regard to different soil pH values.

Kim et al. (2014) had shown that the *Alphaproteobacteria* share decreases with the depth of the soil layer, while other researchers had demonstrated that these bacteria prefer a nutrient rich environment (Nemerguta et al., 2010) resulting from additional nitrogen input (Fierer et al., 2012). Our physicochemical analysis revealed a considerably higher concentration of nitrogen in the soil sample from the bird colony area when compared to the control sample, which is also consisted with previous analysis (Zwolicki et al., 2013), but despite that fact, the share of *Alphaproteobacteria* changed only insignificantly.

Some of bacteria from the *Actinobacteria* phylum are able to efficiently fix nitrogen available in the air and presence of such a functional bacterial group in a soil with permanent deficits in nitrogen is comprehensible. Thus, this group can be of a high importance in the area with the constant lack of nutrition and/or when the high loads of nutrient deposition in the area of the bird colony are not fully used and are washed away by the thawing snow at the end of winter (Skrzypek et al., 2015). Therefore, reduction in the *Actinobacteria* abundance in the soil samples impacted by the bird colony seems only natural in the context of a high amount of guano deposition and in consequence of a high nitrogen content. Koyama et al. (2014) reported that fertilization reduces the abundance of *Actinobacteria* in the soil, at the phylum level, while Kim et al. (2014) had shown that the abundance of *Actinobacteria* can be also related to the soil pH values. In the study presented here, we observe that within the dominant *Actinobacteria* phylum, the *Thermoleophilia*, and *Actinobacteria* classes are more abundant at higher soil pH values (Figure 3; Supplementary Table 1), although the abundance of the whole phylum is lower.

Winsley et al. (2014) analyzed 225 soil samples spanning the Arctic and Antarctic, for the presence of prevalent bacterial candidate division TM7. These phyla were found in nearly half of the tested samples, in the range of 0.05–2.23% in the total microbial community. In our study, the TM7 phyla constituted 0.08% of the total microbial community in the sample in sample from colony, and 0.15% in control sample. The role of these bacteria, as a candidate division, is still unclear since they remain uncultivable, however, they are commonly found in environmental samples.

In this study, despite differences in the soil's physicochemical features, no significant differences were revealed at the phylum level of bacterial structure communities between both tested samples. Similar results were demonstrated for the Ross Sea region of Antarctica in case of the ornithogenic soils collected from land within the Adèlie Penguin rookeries (Aislabie et al., 2009). These results had shown that despite relatively high nutrient levels and high microbial biomass, the bacterial communities of ornithogenic soils were not more diverse than those of mineral soils in the Ross Sea region of Antarctica. Even though the study presented here and (Aislabie et al., 2009) were performed with the use of completely different tools and analyses, and therefore they cannot be directly compared, they arrived at the same conclusion, i.e., that a change in the soil physicochemical features due to vicinity of a seabird colony has no substantial impact on the high taxonomic ranks of bacterial structure itself, although a limited number of phyla may still be impacted. The presence of the little auk colony, which influenced the soil pH and nitrogen distribution, impacted only several of the phyla, like *Actinobacteria* and *Proteobacteria*. Additional analysis, performed at the class level for each phylum, displayed significant differences between the samples for five of the phyla, *inter alia Proteobacteria.* Although, this phylum, together with *Actinobacteria*, was in the most part responsible for the differences between both tested samples, we were able to observe only minor difference at a class level.

Acidification of the soil may have a strong impact on arctic soil bacterial communities, as determined by local environmental factors (Chu et al., 2010). Our results for high taxonomic level analysis suggest that the presence of the little auk colony, which in general had influenced the soil physicochemical features (but mostly nitrogen distribution), impacts only some of the bacterial phyla, for e.g., *Actinobacteria* and *Proteobacteria.* When the analysis was carried out at the class level for each phylum, significant differences between the samples were observed for five phyla: *Actinobacteria, Armatimonadetes, Cyanobacteria, Gemmatimonadetes*, and *Proteobacteria*, wherein *Armatimonadetes* and *Cyanobacteria* accounted together for less than 1% of the total population share. Nevertheless, more analyses should be performed concerning not only a larger number of soil transects and a larger number of the tested samples from this particular colony, but they also should encompass other seabird colonies.

## Author contributions

SZ performed DNA isolation, sequencing data analysis, wrote the manuscript, took part in planning of the study, and discussion; DK performed statistical data analysis, helped in writing the manuscript, and in sample collection; LS, MŁ, and JŁ helped in writing the manuscript and took part in planning of the study and discussions.

### Conflict of interest statement

The authors declare that the research was conducted in the absence of any commercial or financial relationships that could be construed as a potential conflict of interest.

## Supplementary material

Presentation 1 **Detailed taxonomic analyses for different ranks in the two tested soil samples**. Sunburst charts show the relative abundance of the bacterial 16S rRNA gene sequences for each soil sample, at different taxonomic levels. The first level of the sunburst chart represents all phyla present in a particular sample; the next levels represent the class, order, family, and genus, respectively (The most suitable browser is Firefox, html data are available under the following link: https://www.dropbox.com/sh/u6x3dtmoohkkrja/AADQScCONd5lry6KX84JFuxua?dl=0.

Supplementary Figure 1 **Rarefaction analysis of the tested soil samples based on the chao1 measure**.

Supplementary Table 1 **Detailed taxonomic analyses for different ranks in the tested soil samples**. Table shows the relative abundance of bacterial 16S rRNA gene sequences for each tested soil sample (A and C), at different taxonomic levels.

Supplementary Table 2 **Differences between sample A (under the influence of the little auk colony) and sample C (the control sample) at the class level within each phylum, compared by chi-square test analysis**.
