# Supplementary material for: The Arctic Soil Bacterial Communities in the Vicinity of a Little Auk Colony
Source: Front Microbiol. 2016 Sep 9;7:1298. doi: 10.3389/fmicb.2016.01298 (PMC5016516; doi:10.3389/fmicb.2016.01298)
Supplement: Presentation 1 — Detailed taxonomic analyses for different ranks in the two tested soil samples. Sunburst charts show the relative abundance of the bacterial 16S rRNA gene sequences for each soil sample, at different taxonomic levels. The first level of the sunburst chart represents all phyla present in a particular sample; the next levels represent the class, order, family, and genus, respectively (The most suitable browser is Firefox, html data are available under the following link: https://www.dropbox.com/sh/u6x3dtmoohkkrja/AADQScCONd5lry6KX84JFuxua?dl=0. [file Presentation1.ZIP › sample A.html]

Javascript must be enabled to view this page.

members
magnitude

A.krona

0.999999997912818

0.999865363425484

0.0001954372

2.17152436667e-05

2.17152436667e-05

2.17152436667e-05

2.17152436667e-05

5.21165360668e-05

3.90873898668e-05

4.343e-06

4.343e-06

1.30291462e-05

0.0033615197

0

0.0033615197

6.94887310002e-05

4.343e-06

0

1.73721949334e-05

2.17152436667e-05

2.60582924001e-05

0.26007044374834

0.0858490440162699

0.0002475536924001

4.343e-06

2.60582924001e-05

8.6860974667e-06

8.6860974667e-06

0.0012117106442674

0.0001910941442674

4.34304873335e-05

9.55470721337e-05

8.6860974667e-06

0.0010206165

0.0057762547518673

1.73721949334e-05

0.0057241381670671

0

0.0006080268

0.0002345246

0

6.08026822669e-05

0

0.000178065

5.21165848002e-05

0.0070704833

2.60582924001e-05

0.0610111485164008

0.0052203446

0.0040260062

0.0240127163645337

0.014805453120867

9.9890120867e-05

0.0034570667436667

2.17152436667e-05

0.0018284235

0.0020846634

4.343e-06

0.0004777353974667

8.6860974667e-06

0

0.0002388677

0.0061540999924001

0.0041649837

0

0

4.343e-06

0

2.60582924001e-05

1.30291462e-05

1.30291462e-05

6.08026822669e-05

6.08026822669e-05

0

1.30291462e-05

0

0

0

0.0002909843

8.6861e-05

0.0002041233

0.0003083564873334

1.30291462e-05

0

1.73721949334e-05

1.30291462e-05

0.0056980798544005

0.0012421118949334

1.73721949334e-05

0

0.0043734500335335

0

0

0

5.64596335335e-05

0.0012725132645337

0.0012725132645337

0

0

6.94887797336e-05

3.90874386001e-05

0

0.0002388677

1.30291462e-05

7.38318284669e-05

8.6860974667e-06

6.51457310002e-05

0.034032129975868

0.0224057884468006

0.0012030245

0.0003995605

0

2.60582924001e-05

0

0.0024190781

0.0001606928284669

8.6861e-05

7.38318284669e-05

8.25179259336e-05

0

1.30291462e-05

4.77735360668e-05

0.0079651514

0

0.0079651514

4.343e-06

5.21165848002e-05

0.0001129193

0.0001129193

0.0001129193

0

0.0005906546386001

0.0004429909386001

0.0004429909386001

3.90874386001e-05

0.0001476637

8.6860974667e-06

0

5.64596335335e-05

0.0023886768386001

0.0002128094

0.0013116007

3.90874386001e-05

0

0.0082778507493339

0.0010597039

0.0010597039

4.343e-06

4.343e-06

0.0004560201

0

0

0.0007426613

0

0

0

0

0

0.0007426613

0.0007426613

4.343e-06

0.0009337554721337

0.0005602533

0.0001129193

0.0002475537721337

0

9.55470721337e-05

4.343e-06

4.343e-06

0

0

0

0.0049250172772002

0.0035352416386001

0.0002866412

2.17152436667e-05

0

0.00035613

1.73721949334e-05

0

0.0013897756386001

3.90874386001e-05

0

0

0.0001520067

0.0001520067

0.131828901080935

8.686e-06

8.686e-06

4.343e-06

0.0003995604924001

0.0003995604924001

0

0.0001259484

0

0.0001129193

0

2.60582924001e-05

0.0097240861

0

0

0

0.0199476228

0.0060368377

0.0139107851

0.0033658628

0.0002866412

0.0002866412

0.0014679505

0.020407986

0.0400906829

0.0001346345107337

0.0001042331696003

8.25179259336e-05

8.25179259336e-05

0.0098413482778008

2.60582436667e-05

2.17152436667e-05

8.6860974667e-06

4.343e-06

0.0026883472

0.0068272724670671

8.6860974667e-06

0.0002518968

0.0002475538

0.0002258385

5.21165848002e-05

0

0

4.343e-06

0

0.0020238607

8.6860974667e-06

0

0.0001172623

0

4.34304873335e-05

0

0.0002562398797335

4.77735360668e-05

2.17152436667e-05

4.77735360668e-05

4.77735360668e-05

4.77735360668e-05

4.77735360668e-05

0

4.77735360668e-05

0.0005428811

0.0005428811

0.0007860917158004

0.0004820784

9.55470721337e-05

2.60582436667e-05

0

4.343e-06

0.000178065

0.0430135545462

0.0430135545462

0.0430135545462

0.0001954372

0.0001954372

0.0401775437462

0.0022410131

0.0379235015

0.0005298519

0.0021107217

4.343e-06

0.0018457956949334

0.0004039035

0.0008121500949334

0.000178065

8.6860974667e-06

0.0006167129

8.6860974667e-06

0.0003821883

0.0814017621651343

0.0027882372924001

0.0027882372924001

0.0027882372924001

0.0002736121

3.04012924001e-05

4.343e-06

0.035643400920867

0.035643400920867

0

0.035643400920867

5.21165848002e-05

0

0.0012117106

0.0323426839

4.77735360668e-05

0.0123299152797335

0.0123299152797335

0.0041345824

0

0.0001997802

0.000182408

6.94887797335e-05

3.90874386001e-05

0.0001693789

0.0021584952

0.0021584952

0

0

0.0283861664

0.0263058461

0.0263058461

0.0108489357

9.55470721337e-05

5.21165848002e-05

4.34304873335e-05

0.119103768175268

0.0029315579

0.0022801006

0.0142929732772003

0

0

0.0030575063

0.0002736121

0

0.0027838942

4.343e-06

0.0006427711949334

0.0006427711949334

1.73721949334e-05

0.0003387578

0.0067447547

0

6.08026822669e-05

0.0002258385

0.0023669616

0.0002432107

0.0002432107

0.0134677941

1.30291462e-05

0.0492241143

2.60582924001e-05

0.0096936848

0.0020803203

0.0009467846

0.0004169327

0.0005298519

0.0009337555

0.0007774057

0.0114352473

0

0

0

0.0139281572645337

1.30291462e-05

0

0

1.30291462e-05

0.0007774057

0.0130812627848002

0.0130291462

5.64596335335e-05

0.0020108314949334

0.0019891163

4.343e-06

1.73721949334e-05

0.000534195

0.0004082466

0.0001389776

0

0.0001259484

4.34303898667e-05

4.34303898667e-05

4.34303898667e-05

4.343e-06

1.73721462e-05

4.343e-06

0

0

0

0

0

0.0008034639

0.0008034639

0.0007991209

0.0005298519

0

4.343e-06

0.0059977499778006

0.0056633354822667

0

0.0056589924822667

0

0

0

0

0

0

0

1.30291462e-05

0.000264926

0.000264926

0.000264926

8.6860974667e-06

8.6860974667e-06

0

0

0.000442991

0

0

1.73721462e-05

4.343e-06

1.30291462e-05

0.0049119880924

0.0048859298

4.343e-06

1.30291462e-05

0.0003344144955339

4.343e-06

0

0

4.343e-06

4.343e-06

0.0003300714955339

1.73721949334e-05

1.73721949334e-05

0

0.000104233120867

5.64595848002e-05

0

5.21165848002e-05

0

0.0001302914

4.343e-06

0.0001259484

0

0

0

6.51456335335e-05

6.08026335335e-05

4.343e-06

0.0002084663

0.0002084663

0.157461574851736

0.0308703903410021

1.30291462e-05

1.30291462e-05

1.30291462e-05

0

0.0308443320486021

0

0

0.0008382084

0.0008382084

0

0.0002996704

0

0.0002996704

0

4.77735360668e-05

0

0.0024972530873334

4.343e-06

0.0001042332

0

1.30291462e-05

1.30291462e-05

9.1204e-05

0.0001129193

0.0004690493

0.0011899954

0.0011899954

2.17152436667e-05

8.6860974667e-06

0.0002128093924001

0.0002128093924001

2.60582924001e-05

0.0061236987772002

0.0006558004

3.04013411334e-05

3.90874386001e-05

0

0

0.0017024750974667

8.6860974667e-06

0.001072733020867

3.90874386001e-05

6.08026822669e-05

0

1.73721949334e-05

0

0.0027838942

2.60582924001e-05

2.60582924001e-05

9.98900974667e-05

1.30290974667e-05

4.343e-06

8.6860974667e-06

0

0

8.6861e-05

8.6861e-05

0.0003213856

0.0002996703924

0.0002736121

1.30291462e-05

2.17152436667e-05

0

0

0.0004864214696004

0

0

0

8.6860974667e-06

0.0001563498

4.343e-06

4.343e-06

9.55470721337e-05

0

1.73721949334e-05

0

8.686e-06

4.343e-06

4.343e-06

0.0123429443284669

4.343e-06

7.38318284669e-05

0.0001520067

0

0.0080954428

4.343e-06

0

0

0

0.0002128093772003

0.0001346345

0

0.0001520067436667

2.17152436667e-05

0.0042822461

0.0042822461

7.81748772003e-05

7.81748772003e-05

7.81748772003e-05

7.81748772003e-05

0.0571024047

0.0571024047

0.0272222295

0

0.0049727908

0.0049727908

0.0170073788

1.30291462e-05

0.0651153296873335

0.0351439502898668

0.0006992307974667

8.6860974667e-06

0.0027448068

0.0146230450924001

2.60582924001e-05

0.0299713793974667

0.0258498261

8.6860974667e-06

0.0012334258220681

5.21165848002e-05

0

0.0002128093974667

0.0002041233

8.6860974667e-06

8.6860974667e-06

0.0006514573310002

0.0006514573310002

0.0006514573310002

0.0005863116

0.0002345246316007

7.38318284669e-05

6.51457310002e-05

6.51457310002e-05

8.25178772003e-05

8.6860974667e-06

7.38317797336e-05

7.38317797336e-05

4.343e-06

0

0.0012985716544005

0.0001650359

0

0.0001650359

2.60582924001e-05

0

0

0

2.60582924001e-05

2.60582924001e-05

2.60582924001e-05

0

0

0

0

0

0

0

0

0

0

0

0

0

0

0.0003604730620004

6.51457310002e-05

6.51457310002e-05

0

0.0002301816

0.0007470044

0.0001520067

0.0042475016873335

0

0.0005645963

0.00213678

0.0015026949

0.0001389775949334

1.73721949334e-05

0.0001085762

0.0001085762

0.0001085762

0.0311179440904673

0.0002779551

1.30291462e-05

0

0

0.002223641

0.00213678

8.6861e-05

0.0064146829335335

1.73721949334e-05

0.0052898334

0.000182408

1.73721949334e-05

0.0008859819

0

2.17152436667e-05

9.1204e-05

4.343e-06

8.6861e-05

0.0213938580462

0.0100107273

0.0097414583

0.0032225422

0.000269269

0.0094939045

0.0094939045

0.0009250694

0.0017936791

0.0018761971

0.0018761971

0.0018761971

1.30291462e-05

0.0001216053645338

6.08026822669e-05

6.08026822669e-05

0.188470942987934

0.0013680604

7.38318284669e-05

0.0027057194

0.0023843338

0.0023843338

0.0362557707

4.343e-06

0.0362514277

0.0250984786

0.024955158

0

0.0016243002

0

0.0021715244

0.0021715244

2.60582924001e-05

0.0003300717

1.73721949334e-05

1.73721949334e-05

1.73721949334e-05

0.048742036

0.0078696043

0.0408724317

0.0034310085

0.0011986815

0.0181539436924001

0.0095025906

0.0016243002

0.0070009946

6.94887797336e-05

0.0704746519

0.0014896657

0.0001259484

0.0634562851

0.0109401398

0.0052898334

0.0003213857

0.0001650359

0.002944587

0.0010249594

0.0010249594

4.343e-06

0

0.0010206164

4.343e-06

0.0002909843

0.0299279488271345

1.30291462e-05

0.0224361896259336

0.0224361896259336

0.0016156141

0.0205339343259336

4.343e-06

0

6.94887797336e-05

1.30291462e-05

0

0.0010379887310002

0.0010379887310002

0.0004864214974667

0

8.6860974667e-06

0.0004951076

0.0004951076

0.0001563498

5.64596335335e-05

3.04013411334e-05

2.60582924001e-05

0.0042388156462

0.0042388156462

0.0002128094462

0.0001259484462

1.30291462e-05

0.0001433205974667

0.0001433205974667

8.6860974667e-06

8.6860974667e-06

0

0.0020499189828673

0.0020499189828673

0.0020412328854006

2.17152436667e-05

9.9890120867e-05

2.17152436667e-05

3.04013411334e-05

0

4.77735360668e-05

0

0

0

0.0018327666

0.0018327666

0.0014332061

4.343e-06

0.0001302915

0.000264926

0.0003170426

0.0006688294183337

8.6860974667e-06

0.0005559102

4.343e-06

8.25179259336e-05

1.73721949334e-05

2.17152436667e-05

0.0644855873335335

0.0001346345

0.0307096974436667

0.0022844436

0.000538538

0.0112788975436667

2.17152436667e-05

0.0096155099

0.0016416724

0.0016416724

0.0012247397

0.0124298055

0.0329507107

0.0005472241

0.0001085762

0.0001346344873335

9.1204e-05

9.1204e-05

9.1204e-05

4.34304873335e-05

4.34304873335e-05

4.34304873335e-05

4.34304873335e-05

4.34304873335e-05

3.47443898668e-05

8.6860974667e-06

0

0

0

0

0

0
