# Supplementary material for: The Arctic Soil Bacterial Communities in the Vicinity of a Little Auk Colony
Source: Front Microbiol. 2016 Sep 9;7:1298. doi: 10.3389/fmicb.2016.01298 (PMC5016516; doi:10.3389/fmicb.2016.01298)
Supplement: Presentation 1 — Detailed taxonomic analyses for different ranks in the two tested soil samples. Sunburst charts show the relative abundance of the bacterial 16S rRNA gene sequences for each soil sample, at different taxonomic levels. The first level of the sunburst chart represents all phyla present in a particular sample; the next levels represent the class, order, family, and genus, respectively (The most suitable browser is Firefox, html data are available under the following link: https://www.dropbox.com/sh/u6x3dtmoohkkrja/AADQScCONd5lry6KX84JFuxua?dl=0. [file Presentation1.ZIP › sample C.html]

Javascript must be enabled to view this page.

members
magnitude

K.krona

1.00000000158542

0.0004191715205701

1.43306511131e-05

1.43306511131e-05

1.43306511131e-05

1.43306511131e-05

1.43306511131e-05

0.0003905102183439

0.0003905102183439

0.0003905102183439

0.0003905102183439

0.0003905102183439

0.0002937783

2.1496e-05

5.73226044525e-05

1.43306511131e-05

1.43306511131e-05

1.43306511131e-05

0.999580830064848

3.58266277828e-06

3.58266277828e-06

3.58266277828e-06

3.58266277828e-06

3.58266277828e-06

0.00400183438174671

0.00196329915725794

2.1496e-05

0

0

2.1496e-05

2.1496e-05

0.00194180315725794

0.0007523591

0.0005911393

2.1496e-05

0.000265117

3.58266277828e-05

0.0006198006811222

7.88185811222e-05

0

0.0004227542022262

1.43306511131e-05

0.0004084235511131

1.43306511131e-05

7.16532555656e-06

3.58266277828e-06

8.598390667878e-05

8.598390667878e-05

3.58266277828e-06

0.00203853522448877

0.00193822066669687

0.00015405446277828

0.0001504718

0

7.16532555657e-06

2.1496e-05

2.1496e-05

1.43306511131e-05

1.43306511131e-05

0.0001540545

0.0001970465

0.0001755505

0.0001755505

3.58266277828e-06

3.58266277828e-06

3.58266277828e-06

3.58266277828e-06

0.00118227876277828

0.0011786961

0

0

3.58266277828e-06

3.58266277828e-06

9.67318950136e-05

2.1496e-05

2.1496e-05

1.79133138914e-05

0.0022893215905611

0.0022893215905611

3.94092905611e-05

0.0003976756

0.0012360187

0.000616218

0.144868551963937

0.0115397567833485

0.0001182279

0.0114215288833485

0.0016551902

0.0001648025

0.0050766331555657

0.0050049799

3.58266277828e-05

0.000286613

0.000286613

3.58266277828e-06

0.0032673885

0.0032673885

0.0012682626

0.0014044038

0.0007308632

0.0004263369

0.0005302341

6.09052672308e-05

0.0014473958

0.0014473958

0.0014258998

0.0004406675

2.1496e-05

1.0748e-05

0.0459727287

0.0061657626

0.0398069661

0.0020958577

0.0026869971

0

0

0

0.0629545503694571

8.95665694571e-05

0.0525970723

0.0069826098

0.008881421

0.0012181053

0.0146029333811222

0.0035862454

0.0108160589

0.0001218105

0.0001648025

0.0001325585

0.0004370849

0.0033927817

0.0027156584

0.0006771233

0.00010747988334848

0

7.52359183439e-05

2.86613022263e-05

0

3.58266277828e-06

0.0419279023850409

0.0001433065

3.2244e-05

0.0001110625

0.0043314392811222

0.0043314392811222

0.0043314392811222

0.0017805834

7.88185811222e-05

0

0.0076955596

0.0076955596

0.0004872421

0.0002937783

0.0008705871

0.0001289759

1.0748e-05

0.0001146452

0.0005768087

0.0023502268277828

3.58266277828e-05

3.58266277828e-05

0.0023144002

0.0184901225439005

0.0184901225439005

0.0184363826022263

0.0108124763

0.0001898811

2.86613022263e-05

0.0002901957

0.0040770702

5.37399416742e-05

0.0089172476322354

0.0088240984

0.0088240984

0.0069037912

0.0001182279416742

4.2992e-05

0

4.2992e-05

0

2.1496e-05

3.94092905611e-05

0.00044783282778287

0.00031169162778287

7.16532555657e-06

2.86613022263e-05

0.0001361412

5.0157278896e-05

0.0001468892161177

0.0001003146

4.65746161177e-05

0

0

7.16532555657e-06

0.0016910167811222

0.0016910167811222

0

0.0016910167811222

0.0008347604

7.88185811222e-05

0

0

0

0

0.00147605707666967

0.0001648025

2.1496e-05

1.0748e-05

0.0003869276

0.000902831

1.43306511131e-05

1.0748e-05

3.2244e-05

2.1496e-05

6.09052672308e-05

6.09052672308e-05

6.09052672308e-05

0

0

0

0.332768467038471

0.0210803878

0.0206755469

1.0748e-05

0.161495690057792

0.0865786287

0.0790550369

0

0.074917061357792

0.0093686632

0.0034142776022263

2.86613022263e-05

0.0297289357555657

7.16532555657e-05

0.0991860191762265

7.16532555657e-06

0

0

0

0.09915735785067

3.224396500457e-05

3.224396500457e-05

2.5078639448e-05

5.373997666967e-05

7.16532555657e-06

1.43306511131e-05

0.0015656236

0.00467537499056108

0.0046502963277828

3.58266277828e-05

3.58266277828e-06

3.58266511131e-05

2.1496e-05

7.16532555657e-06

7.16532555657e-06

7.16532555657e-06

0.0002257078044525

5.73226044525e-05

0.00078818579946618

2.5078639448e-05

0

0

0

3.58266277828e-06

1.0748e-05

1.0748e-05

0

7.16532555657e-05

0

0.0005087381

5.37399416742e-05

0

0

0.0027264063205702

3.94092905611e-05

0.0001397238

0.0001397238

0.0015333797

3.58266277828e-05

2.86613022263e-05

0.0001898811

2.1496e-05

2.1496e-05

0.000913579

3.2244e-05

0.0002006291

0.0002006291

0.00100672826277828

0.0004227542

3.58266277828e-06

7.16532555657e-06

7.16532555657e-06

0.0001827158511131

1.43306511131e-05

0.0458509182833484

0.035973517

0.0004048408511131

1.43306511131e-05

1.43306511131e-05

7.88185811222e-05

0

0.0103467299855747

2.5078639448e-05

0.0026440051044525

5.73226044525e-05

0.0001218105

0.000275865

1.0748e-05

5.37399416742e-05

0.00084909111389138

1.43306511131e-05

1.43306511131e-05

0

1.0748e-05

3.58266277828e-06

0.00352892286277828

0.00352892286277828

3.58266277828e-06

0.0002364558

0

1.0748e-05

8.95665694571e-05

2.5078639448e-05

2.5078639448e-05

0.0179276445627783

2.1496e-05

0.00103897216277828

3.58266277828e-06

0.0053489155

0.0003475182905611

0.0003475182905611

0.0003475182905611

0.000308109

0.0506481037138914

0.0506481037138914

0.0191278366

0.0014545611

0.0014545611

0.021205781

1.79133138914e-05

0.154681466306418

0.0056355286333939

0.0001898811277828

0.0001540545

0.0001540545

0

3.58266277828e-05

7.16532555657e-06

7.16532555657e-06

0.00338203360391875

0.00198837780391875

0

6.09052672308e-05

7.165325556568e-05

3.58266277828e-06

7.16532555657e-05

7.16532555657e-06

0.0013936558

0.0001504718

6.807059278733e-05

3.224396500457e-05

3.224396500457e-05

7.16532555657e-06

3.582662778276e-05

2.149597666966e-05

3.58266277828e-06

3.58266277828e-06

1.43306511131e-05

1.0748e-05

0

0

0.0002149598

0.0002149598

1.0748e-05

1.0748e-05

0.00070936726277828

3.58266277828e-06

0.00094582302555657

0.0004836595

0.0002579517

0.00030452632555657

7.16532555657e-06

0.0001253932

0.0001325585

0.0001325585

0

0.074934975051204

0.0016480249

0.00961228428334845

0.00934358455779188

0.00187015

1.0748e-05

0.0005983047

0.0004621634511131

1.43306511131e-05

8.24012439005e-05

0.0004549982

3.58266277828e-06

7.16532555657e-06

0.0005051555

0.00083476039278738

6.807059278738e-05

3.58266277828e-06

3.58266277828e-06

0.0003833449

0.00167668626001816

0.00167668626001816

0.00014688916277828

3.58266277828e-06

4.2992e-05

0.000967319

2.866130222628e-05

2.5078639448e-05

9.67318950136e-05

0.00919311271611767

0.0062624945905611

1.0748e-05

1.0748e-05

3.94092905611e-05

1.0748e-05

0.00185581932555657

0

7.16532555657e-06

0.0017805835

0.000988815

0

0.000318857

0.0002615344

0.0007917685

0.0479467760989323

0.0004872421905611

0.0001289759

0.000318857

0.003432191

0.0023896361

0.000171967839448

1.0748e-05

2.5078639448e-05

0

0.00055173008833485

1.074798833485e-05

3.58266277828e-06

0

0.0001468892

0.0004120062

0.0004120062

0.0259313131511131

0.0181569349255566

7.16532555657e-06

0.0015548756

0.00176267012555657

7.16532555657e-06

0.0032351445

0.00566418981389138

3.58266277828e-06

1.0748e-05

1.0748e-05

1.43306511131e-05

1.43306511131e-05

0.0044639978

8.95665811222e-05

1.0748e-05

8.95665694571e-05

1.79133138914e-05

1.79133138914e-05

0.00032602235111314

7.16532555657e-06

0.0003116917

0.0451522993100637

0.0041738021

0.0009279097161177

0.0004908248161177

4.65746161177e-05

4.65746161177e-05

0.0002901957

0.0011536174

0.00181641

4.2992e-05

4.2992e-05

0.0116579847

1.0748e-05

1.0748e-05

1.0748e-05

0.00907488505503182

0.0004012583

2.1496e-05

2.1496e-05

0.0018199927

0.00638788787613582

0.0008741697138914

0.0008562564

0.0003116917

0.0003045263

6.090526723078e-05

3.58266277828e-06

0.000340353

1.0748e-05

2.86613022263e-05

5.73226044525e-05

3.58266277828e-06

3.58266277828e-06

0.0007308632

3.58266277828e-06

0.0002615344

4.2992e-05

0.000154054478896

0

5.0157278896e-05

0

0

0

0

0.00035110091335764

0.00035110091335764

2.5078639448e-05

0

7.16532555657e-06

3.94092905611e-05

7.16532555657e-06

9.31492322354e-05

0.00284821692555657

0.0012252707

0.0016157809

0.0287329555795206

0.0067855633416742

0.0067318234

5.37399416742e-05

3.58266277828e-06

0.00105330276277828

3.58266277828e-06

0.0001038972

0.0004872421

0.0004764941

0.0004764941

7.16532555657e-05

0.0006090526277828

0.0006090526277828

0.000573226

0.0007022019

0.0001361412

0.0005660607

0.0005660607

0

7.52359183439e-05

2.86613022263e-05

0.018533114757258

7.16532555657e-06

0.0007236979

0.0001468891739096

5.73226044525e-05

8.95665694571e-05

0.00041200626723077

7.16532555657e-06

5.37399416742e-05

0.0049118307

3.2244e-05

3.94092905611e-05

0.0005803914

0.0001289759

1.43306511131e-05

0.0003439356

0.0003439356

0.0003439356

0.15771598065895

1.79133138914e-05

0.0326667191833485

0.0008849177

8.24012439005e-05

2.507863944797e-05

7.16532555657e-06

1.79133138914e-05

1.79133138914e-05

0.0316743216

0.0315704244

0.0039480944

1.0748e-05

0

0.0037510479

0.0019418032

0.0013757425

0.0054492301

0.0068536339044525

0.0022893215044525

0.0012037747

0.0010282242

0.0041308102

0.0035360882

0.0087667758

0.000286613

0.0254870629016924

0.005832575

0.0047255322

0

0.0012718452672308

0.0012718452672308

6.09052672308e-05

7.16532555657e-06

7.16532555657e-06

0.0007810205

9.31492322354e-05

0.0053095062

0.0001253932

0.0042347073511131

1.43306511131e-05

0.0036256547

0.000594722

0.0020170391

0.0020170391

0.0108375549

3.58266277828e-06

3.58266277828e-06

3.58266277828e-06

0.0613889267

0.0019346378950136

0.0005373994

0.0011213734

0.0001576372

2.1496e-05

0.00219258968334845

0.0002794477

8.956656945707e-05

7.16532555657e-06

0

0

0.0002507864

1.0748e-05

1.0748e-05

0.0002400384

0.0014438131511131

0.0014438131511131

0.0014294825

0.0010318069

3.58266277828e-06

0.0001253932

0.0001038972

0.0001038972

0

2.1496e-05

0.0172648518322354

0.0172648518322354

0.0172648518322354

3.58266277828e-06

3.58266277828e-06

0.0122132973

0.0006126353

0.0103825567

0.0049584053

8.95665694571e-05

0

0.0004227542

0.0004227542

0.0301624381872671

0.00131125462057018

0.00131125462057018

0.0002292904439005

2.86613022263e-05

5.37399416742e-05

0.0010604682

0.0010604682

0.0001038972

2.149597666968e-05

3.58266277828e-06

0

0.0001791331

0.0188698848672308

0.0188698848672308

0.0166092245672308

3.58266277828e-06

0.0001110625

0

0.0002686997

5.73226044525e-05

0.0019489686

3.58266277828e-06

3.58266277828e-06

3.58266277828e-06

3.58266277828e-06

0.0044245886577919

0.0044245886577919

0.0043529353905611

3.94092905611e-05

1.0748e-05

0

0.0003009437

4.2992e-05

0.0001683852

3.5826639448e-05

2.5078639448e-05

1.0748e-05

0.0001576372

0.005216357078896

0.005216357078896

0.000458580878896

5.0157278896e-05

0

0.00586840179225342

5.732260445247e-05

2.149597666967e-05

2.149597666967e-05

2.149597666967e-05

1.43306511131e-05

3.58266277828e-05

3.58266277828e-05

3.58266277828e-05

0.0025687691811222

0.0003654315811222

7.88185811222e-05

0.001805662

0.0003976756

0.0017626701672308

0.0017626701672308

0.0017626701672308

6.09052672308e-05

0.0017017649

0.0006771233

0.0004335022

0.00067712331389138

0.00067712331389138

0.00067712331389138

0.0003690143

3.58266277828e-06

3.58266277828e-06

1.43306511131e-05

0.00032602232555657

7.16532555657e-06

0.000318857

0.0616432959039187

1.43306511131e-05

0.0475383524

0.0131017978600182

0.0046538789

0.0028947915600182

0.0009064137

0.0009064137

5.73226044525e-05

0.001859402

0.0019167246

0.0008885004

0.0007057846

0.0007344459

6.80705927874e-05

0.001970464539448

0.0003439356

0.000884917739448

0.0003367703

0

0.0005230688

2.5078639448e-05

0.0004872421

5.37399416742e-05

0.0002042118277828

0.0002042118277828

0.0002042118277828

0.0002042118277828

0.0001683852

3.58266277828e-05

0.0309936157828146

5.37399416742e-05

0

5.37399416742e-05

0.0012073573

0.0002579517

0.0009494056

0.0211986157

0.001497553

0.001497553

0.001497553

0.0130337272

0.0011679481

0.0118657791

0.0040125823

0.0001182279

0.0065491076

0.0065491076

0.0006878713

0.0009064137

7.16532555657e-06

0.00666733553446158

4.65746161177e-05

0.0009816496511131

1.43306511131e-05

0.0052557663

5.73226044525e-05

3.58266277828e-06

0.0003224397

0.0001218105

0.0002901957

0.0001182279

0.0012503493

0.0010962948

0.0001540545

6.80705927874e-05

6.80705927874e-05
