# Supplementary figures and images for: The Arctic Soil Bacterial Communities in the Vicinity of a Little Auk Colony
Source: Front Microbiol. 2016 Sep 9;7:1298. doi: 10.3389/fmicb.2016.01298 (PMC5016516; doi:10.3389/fmicb.2016.01298)

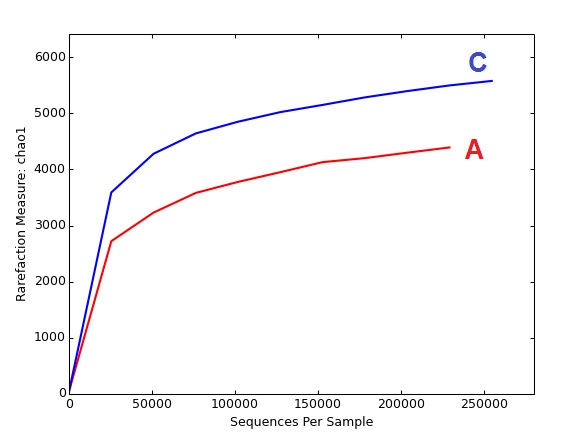

Supplement: Supplementary Figure 1 — Rarefaction analysis of the tested soil samples based on the chao1 measure. [file Image1.jpeg]
