# Supplementary material for: The Arctic Soil Bacterial Communities in the Vicinity of a Little Auk Colony
Source: Front Microbiol. 2016 Sep 9;7:1298. doi: 10.3389/fmicb.2016.01298 (PMC5016516; doi:10.3389/fmicb.2016.01298)
Supplement: Supplementary Table 2 — Differences between sample A (under the influence of the little auk colony) and sample C (the control sample) at the class level within each phylum, compared by chi-square test analysis. [file Table2.DOCX]

| Phylum | χ^2^ | df | p |
| --- | --- | --- | --- |
| *Acidobacteria* | 7.62 | 14 | 0.908 |
| *Actinobacteria* | 12.75 | 5 | 0.026 |
| *Armatimonadetes* | 16.61 | 4 | 0.002 |
| *Bacteroidetes* | 5.32 | 6 | 0.504 |
| *Chlorobi* | 0.87 | 3 | 0.832 |
| *Chloroflexi* | 9.46 | 10 | 0.489 |
| *Cyanobacteria* | 36.76 | 3 | <0.0001 |
| *Gemmatimonadetes* | 15.77 | 5 | 0.008 |
| OD1 | 5.31 | 3 | 0.150 |
| *Planctomycetes* | 5.28 | 7 | 0.625 |
| *Proteobacteria* | 9.72 | 4 | 0.045 |
| *Verrucomicrobia* | 8.24 | 5 | 0.144 |
|  |  |  |  |
